# Orphan Drug Prices and Epidemiology of Rare Diseases: A Cross-Sectional Study in Italy in the Years 2014–2019

**DOI:** 10.3389/fmed.2022.820757

**Published:** 2022-02-17

**Authors:** Federico Villa, Aurora Di Filippo, Andrea Pierantozzi, Armando Genazzani, Antonio Addis, Gianluca Trifirò, Agnese Cangini, Giovanni Tafuri, Daniela Settesoldi, Francesco Trotta

**Affiliations:** ^1^Università del Piemonte Orientale, Dipartimento di Scienze del Farmaco, Novara, Italy; ^2^Agenzia Italiana del Farmaco (AIFA), Rome, Italy; ^3^Dipartimento Epidemiologia Servizio Sanitario Regione Lazio, Rome, Italy; ^4^Department of Diagnostics and Public Health, University of Verona, Verona, Italy; ^5^Zorginstituut Nederland, Diemen, Netherlands

**Keywords:** orphan drugs, HTA, drugs pricing, rare disease, drug coverage, AIFA, Italy, market access

## Abstract

**Introduction:**

It is well acknowledged that the price of orphan drugs is normally higher than that resulting from the value-based pricing. A correlation between the cost of therapy for orphan drugs and the epidemiology (prevalence and incidence) of the related rare disease can be hypothesized.

**Methods:**

This analysis includes all approved orphan drugs by European Medicines Agency whose reimbursement was granted for the first therapeutic indication in the years 2014–2019 in Italy. Regression and correlation analyses were performed to analyze the possible correlations between the logarithm of the annual therapy cost and the epidemiology of the rare diseases, between orphan drugs consumption and epidemiology of related rare disease and between therapy cost and the consumption.

**Results:**

The regression analysis between the annual cost of therapy estimated on the published ex-factory price and the prevalence showed a slightly decreasing, not statistically significant, trend (coefficient: −0.10, *p-*value: 0.41). The results were similar when using the price resulting from the application of Managed Entry Agreements (coefficient: −0.11, *p-*value: 0.40). The regression analysis between sales volume and prevalence showed a positive slope without an acceptable level of significance (*p-*value: 0.04). The correlation analysis between the therapy cost and the sales volume highlighted again an absence of significant association, similarly if considering only ATC L orphan drugs, or the incidence.

**Discussion:**

The definition of the price of an orphan drug seems not to depend on the rarity of the disease, and sales volumes do not correlate with the epidemiology of the rare disease and with the annual cost of therapy.

## Introduction

In Europe, to receive an orphan designation by the European Medicines Agency (EMA), a drug must be intended for use in the treatment of a life-threatening or chronically debilitating disease with a EU prevalence being no more than 5 in 10,000 inhabitants (<250,000 patients, based on EU population of 514 millions) and for which there are no other alternatives or, eventually, the drug represents a significant benefit over the existing therapeutic options. Alternatively, regardless of disease prevalence, manufacturers need to show that the product market would unlikely generate sufficient revenues justifying investments required for its development (1).

The current European legislation requires orphan medicinal products to have access to the centralized marketing authorization procedure in order to ensure early market access [with accelerated assessment programs (2)], as well as several economic incentives to compensate for the potentially low profitability of a medicinal product intended for a limited population of patients (3).

With such a designation, manufacturers receive market exclusivity for 10 years (plus two more years if the orphan indication is extended to the pediatric population) in all EU countries and, in addition, financial incentives for research, development, scientific advice, marketing authorization application, inspections, post-authorization activities as well as market access in individual Member States.

In Italy, the EU orphan designation provides additional benefits during the national pricing and reimbursement (P&R) negotiation. Manufacturers can submit their dossier immediately (instead of 3 months later, as usual, when regulatory approval is ratified by the European Commission), after a positive Committee for Medicinal Products for Human Use (CHMP) opinion, and be granted priority in the procedure for P&R decisions by the Italian Medicines Agency (AIFA).

In addition, orphan drugs are excluded from the refund in case of a breach of the statutory pharmaceutical expenditure ceiling. Once the negotiation process is complete (with a fast track procedure for the definition of P&R), the drug can have direct access to regional drug formularies (if present in the regional system) (3, 4). In addition, under specific circumstances, AIFA can also reimburse orphan drugs prior to their regulatory approval and, within a specific program for independent research, the Agency can fund non-profit research in the field of orphan drugs and rare diseases (4, 5).

In Italy for orphan drugs the same Health Technology Assessment HTA and P&R rules are applied as for non-orphan drugs (6). In 2020 in Italy 82% of orphan drugs authorized by EMA have been commercialized and reimbursed by the Italian National Health Service (INHS). The orphan drugs' spending, being 1.4 billion euro, has represented a 6.0% share of the total public pharmaceutical expenditure (4).

The implementation of the European legislation and the many incentives granted to orphan drugs manufacturers by individual Member States have greatly contributed to massive investments in the field of rare diseases and the successful development of several new compounds (7). Furthermore, following the orphan medicinal products Regulation implementation in EU (8) a rise in the number of scientific publications on rare diseases has been observed. Orphan drugs research activity in Europe has increased considerably compared to the past; during the first decade following the implementation of the Regulation the number of new biotech companies devoted to orphan medicinal products development across Europe has grown by 30% (9). In the period 2000–2020, over 2,382 orphan designations have been issued by the European Commission of which 190 have resulted in authorized medicinal products (10).

In Italy, the upward trend of clinical trials of patients with rare diseases continued significantly in 2018, representing 31.5% of the total (25.5% in 2017), of which almost 80% are for profit trials; there is still an increase in phase I trials on rare diseases (33.7%) and the percentage of trials with advanced therapy products in rare diseases is significant (11%, compared to a global increase in trials with advanced therapy products equal to 4.7%) (11).

By 2024, orphan drugs are expected to reach $242bn, capturing one-fifth of worldwide prescription sales, and orphan drug sales in the world are expected to grow at a Compound Annual Growth Rate (12) of 12.3% from 2019 to 2024, which is approximately double the rate foreseen for the non-orphan drugs market. Orphan drugs are forecasted to be 20.3% of worldwide prescription sales by 2024, with the majority being represented by oncological drugs and cell-and gene-based therapeutics (13).

Despite the benefits from a public health perspective, orphan drugs pose challenges and concerns in terms of sustainability. It has been observed that top 100 US orphan drugs has a mean cost per patient being almost 4.5 times greater than the non-orphan drug cost in 2018, although the difference has diminished in the 2014–2018 time period (13). Although developing a drug intended to treat a rare disease was not often considered profitable as for other medicinal products (14), the actual scenario shows different reality: median cost per patient differential is 5.5 times higher for orphan drugs compared to non-orphan (13).

However, it is well acknowledged that the price of orphan drugs is normally higher than that resulting from the value-based pricing, due to the small markets associated with rare diseases, which necessitate high prices to get returns on investments (15). The literature has found a significant variability on reimbursement and prices across EU countries (16, 17).

A correlation between therapy cost of orphan drugs and prevalence/incidence of the related rare disease can be hypothesized (18). However, these correlations have never been fully investigated.

Objective of this study is to assess if there is a correlation between: (a) the annual therapy cost of orphan drugs with the epidemiology (prevalence and incidence) of the relative rare diseases; (b) the epidemiology of the rare disease and the sales volume of orphan drugs; (c) the therapy cost and the sales volume of orphan drugs in the first year of marketing.

## Methods

This analysis included all EMA-approved orphan drugs whose P&R process for the first therapeutic indication was concluded in the period 2014–2019 with a positive decision on reimbursement in Italy. A longer time period was not considered since it was not possible to gather data through AIFA's electronic information system prior to 2014.

With regards to pricing, if the company (the Marketing Authorization Holder—MAH) presented different prices per unit (different price per pack), the highest one was chosen. We used the ex-factory public price published on the Italian Official Gazette (IOG) -that does not include confidential discounts and the effect of Managed Entry Agreements (MEAs). We also provided the same correlation analyses using the final reimbursed price (FP) which stems from confidential discounts and the estimated financial impact of MEAs (19). The Java program “Plot Digitizer” was used to estimate financial impact of financial-based and outcome-based MEAs.

To standardize the therapy cost of the medicines analyzed we considered the annual therapy cost, based on the dosing regimen reported in the Summary of Product Characteristics (SmPC), including loading dose. If more than one dosage was possible, an average was taken. We considered the single administration for medicines with a one-shot administration (i.e., Nexobrid®).

As for correlation analysis with sales volumes, we excluded drugs whose sales data (at the time of the analysis) were absent (e.g., burosumab, panobinostat).

Prevalence and incidence data were collected according to the following hierarchical sources of information: (i) P&R dossiers submitted to AIFA by the MAHs and stored in the AIFA information systems; (ii) European Public Assessment Reports (EPAR) published on the EMA's official website (20); or information provided by the sponsor and knowledge from the Committee for Orphan Medicinal Products (COMP) at the time of designation (21); (iii) portal for rare diseases and orphan drugs Orphanet; (iv) scientific literature databases (i.e., Pubmed). The attempt to use the same source for prevalence and incidence data (P&R dossiers provided by the companies) and only if missing, to search for information in other sources, made it possible to limit the problems arising from the heterogeneity of information in the literature. Anyway, depending on the source, prevalence (or birth prevalence) and incidence data could be related to Italy, the entire EU, United States or other countries.

Sales data of the first 12 months following commercialization in Italy were collected through an information flow, the so called “Traceability of medicines” flow (4), powered by manufacturers with data on medicinal products purchased by the INHS.

The Shapiro-Wilk test (a test for normal distribution exhibiting high power and able to deal with a small number of observations) was conducted to evaluate the normality of distribution on each variable; logarithmic transformation was used to normalize results.

The available information flow did not allow to obtain consumption data by indication. Therefore, for drugs with more than one authorized therapeutic indication, the total consumption of the molecule was considered. For all continuous variables, the mean plus standard deviation as well as the median and the percentiles were estimated.

As a preliminary analysis, a correlation matrix was constructed to evaluate possible collinearities among the regressors, as well as the strength of the linear associations between the cost of therapy and each variable analyzed.

Regression analyses were conducted to analyze the possible correlation between the logarithm of the annual therapy cost and the prevalence. Subsequently, in a sensitivity analysis, the incidence was considered. Moreover, the association between orphan drugs consumption and prevalence and incidence of related rare disease was investigated through a regression analysis. Lastly, a linear correlation model was done to assess the potential association between the logarithm of the annual therapy cost and orphan drugs consumption (packs).

In a sensitivity analysis, the same associations were investigated considering only orphan drugs belonging to antineoplastic and immunomodulating agents (ATC L).

The data of prevalence and incidence were validated with a Pearson's product-moment correlation analysis, comparing the data provided by industries in the P&R dossiers with the data of EPARs/COMP and Orphanet.

The overall *R*^2^ and the statistical significance of the *p-*value for each regression coefficient were evaluated.

The overall *R*^2^ and *p-*values were tested to evaluate the statistical significance of the analyses.

A *p-*value < 0.05 was fixed as cut-off level to reject the null hypothesis.

All the analyses were performed with SAS 9.4.

## Results

**Figure 1:**
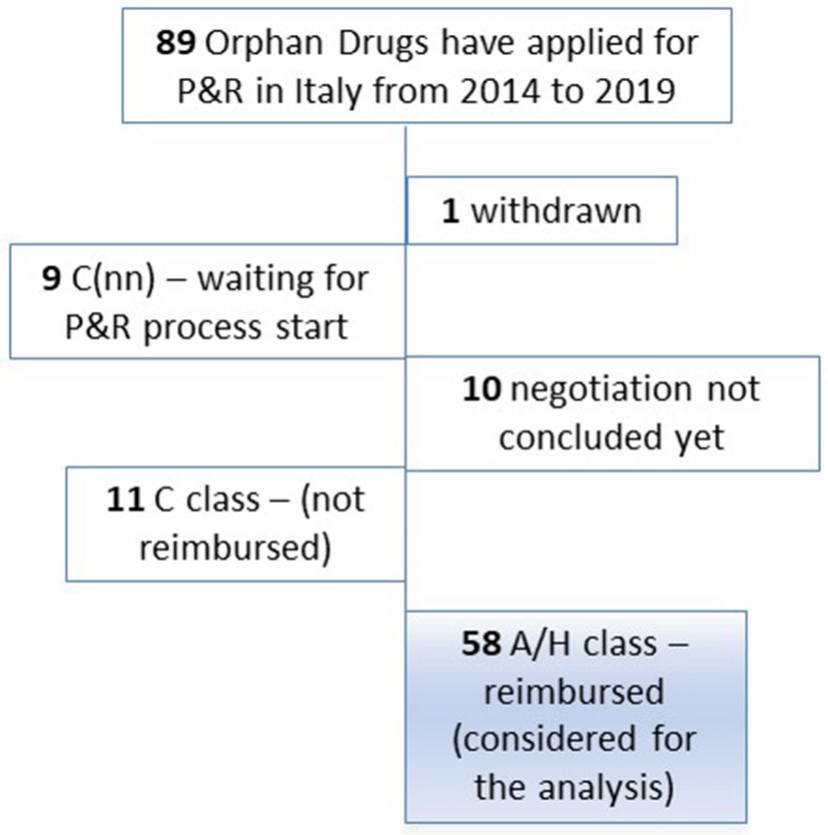
Orphan drugs that applied for P&R in Italy in the period 2014–2019. Legend: A Class: Medicines reimbursed by the NHS. H Class: Medicines reimbursed by the NHS for inpatient use. C Class: Medicines not reimbursed by the NHS. C(nn) Class: Medicines waiting for P&R process, not reimbursed by the NHS.

Out of 89 orphan drugs which have applied for P&R in Italy during the 2014–2019 period, the analysis included 58 drugs which were granted the reimbursability (A/H class; Figure 1). The majority of drugs (31 out of 58; 53.4%) belonged to antineoplastic and immunomodulating agents (ATC L). A share of 81.2% concluded the negotiation process in the 2016–2019 period (56 out of 69 products which have concluded the negotiation during the study period; Supplementary Tables 1, 2).

### Regression Analyses Between the Logarithm of the Annual Cost of Therapy and the Prevalence and Incidence of the Disease

**Figure 2:**
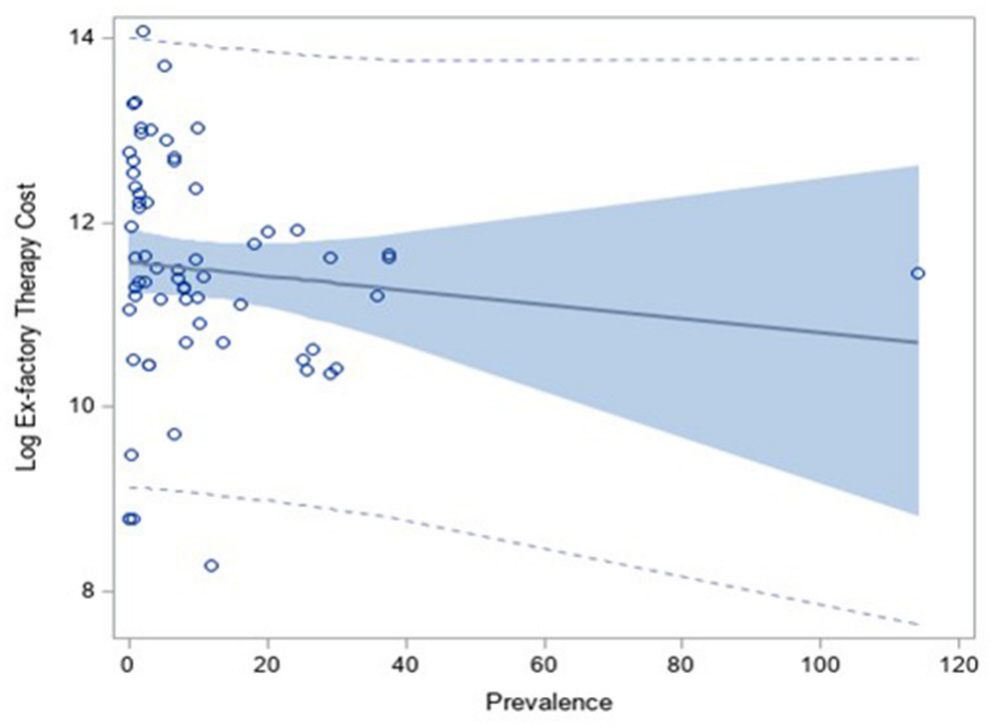
Correlation analysis between the log of the annual therapy cost (calculated on the ex-factory price) and the prevalence of the disease.

The regression analysis between the logarithm of the annual cost of therapy calculated on ex-factory price published on the IOG and the prevalence showed a slightly decreasing, not statistically significant, trend (coefficient: −0.10, *p*-value: 0.41) (Figure 2).

**Figure 3:**
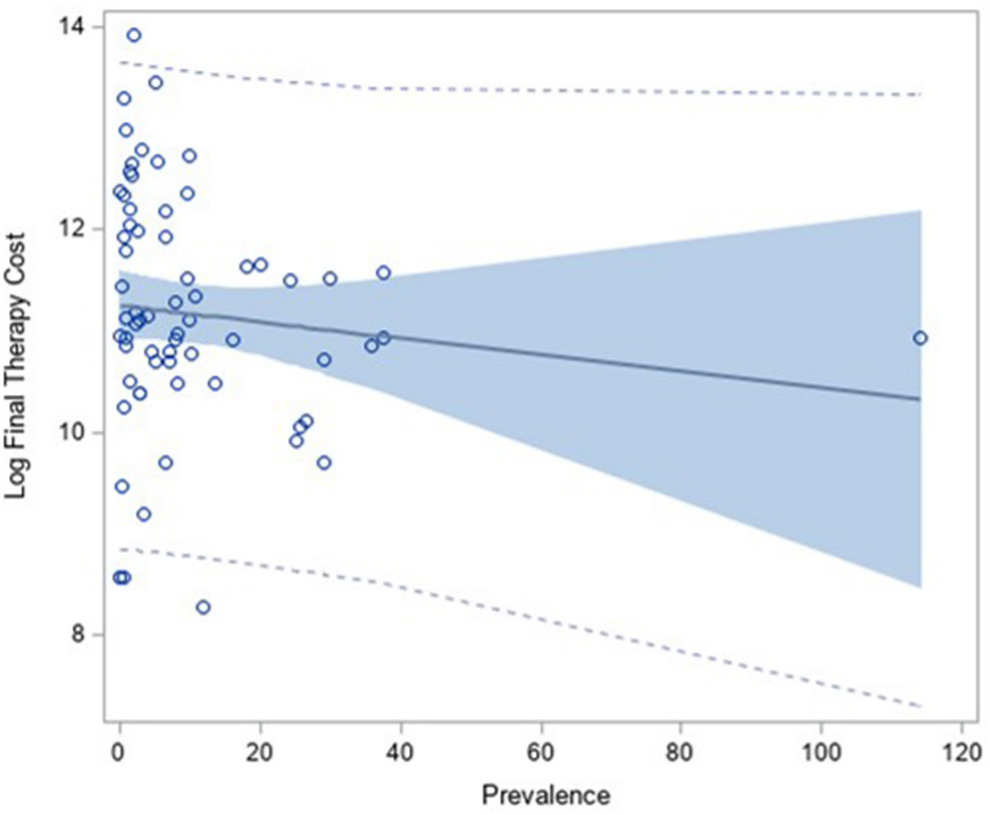
Correlation analysis between the log of the annual therapy cost (calculated on the Final Price) and the prevalence of the disease.

When considering the final reimbursed price (FP) in order to estimate the therapy cost, instead of the price published on IOG, the result was the same: the regression analysis between the logarithm of the annual cost of therapy calculated on the FP and the prevalence presented a slightly decreasing, not statistically significant, trend (coefficient: −0.11, *p*-value: 0.40) (Figure 3).

Indeed, the correlation coefficient between therapy-cost calculated on ex-factory price (published on IOG) and the therapy-cost calculated on the final price (FP) is 0.96, which means that using one price or the other one for the calculation of the annual therapy cost was similar (Supplementary Figure 1). Therefore, the application of the MEAs did not improve the correlation with the prevalence.

When considering, in a sensitivity analysis, the incidence values, similar results were obtained: when the incidence was correlated with the logarithm of the annual therapy cost, calculated on the ex-factory price published on IOG, the regression coefficient was −0.19 (negative but not statistically significant, *p-*value: 0.11). As in the correlation analysis with prevalence, substantial differences were not observed when the logarithm of the annual therapy cost, calculated on the FP, was considered: the regression coefficient was −0.18 (negative but not statistically significant, *p-*value: 0.49) (see Supplementary Figures 2, 3).

### Regression Analyses Between the Prevalence and Incidence of the Rare Disease and the Sales Volume of Orphan Drugs

**Figure 4:**
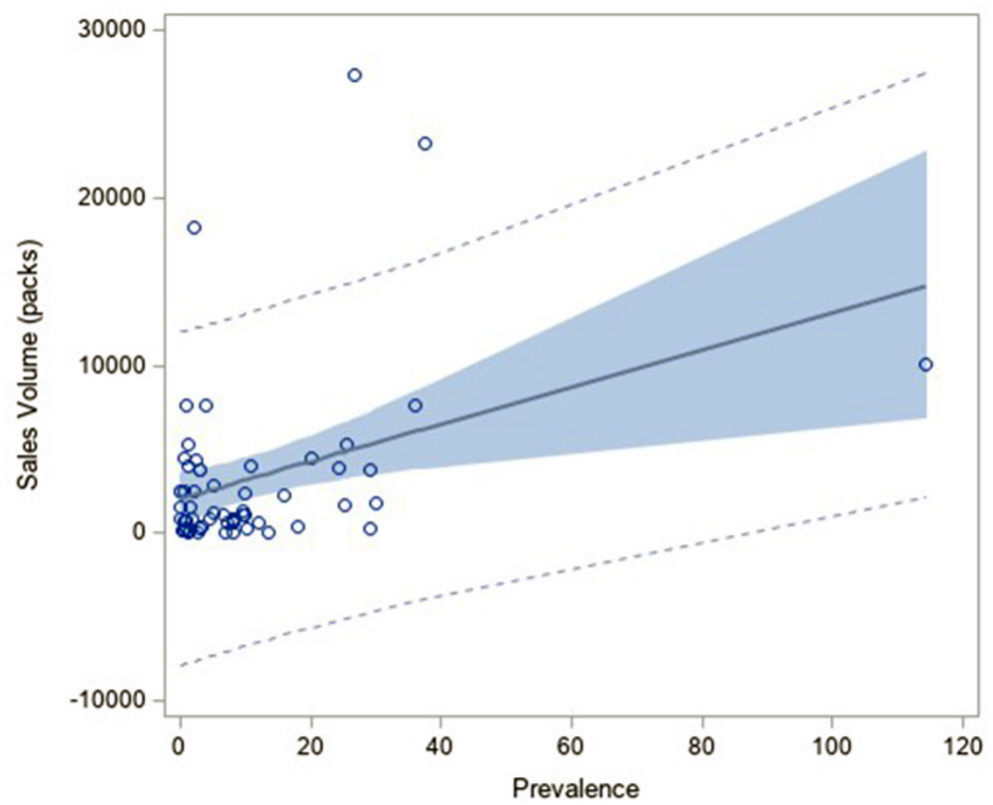
Correlation analysis between the sales volume in the first year of commercialization in Italy and the prevalence of the related rare diseases.

The regression analysis between sales volume in the first year of commercialization and prevalence showed, as expected, a positive slope (regression coefficient: 0.37) but it did not reach an acceptable level of significance (*p-*value: 0.04). It is justified by the fact that the volumes observed included all the indications of the medicines, orphan and eventually not orphan, therefore, they did not follow the prevalence trend (Figure 4).

Considering the incidence led to similar results: when the incidence was correlated with sales volume the regression coefficient was 0.16 (positive but statistically not significant, *p*-value 0.24) (see Supplementary Figure 4).

### The Correlation Analysis Between the Logarithm of the Therapy Cost and the Sales Volume

The correlation analysis between the logarithm of the therapy cost, calculated on the FP, and the sales volume showed again an absence of statistically significant association: the regression coefficient was close to zero (0.08) while the intercept was positive as expected (11.14), which means that the annual therapy cost was totally independent from consumptions (see Supplementary Figure 5).

The analysis did not identify any statistical predictors among those considered (i.e., prevalence, incidence, volume sales) being able to explain the variability and distribution of the annual therapy cost of each treatment (specifically the logarithm of the cost).

### Sensitivity Analysis on Antineoplastic and Immunomodulating Agents (ATC L)

The sensitivity analysis which considered only orphan drugs with ATC L confirmed results obtained for the entire sample of products considered in the study: the regression analysis between the logarithm of the annual therapy cost, calculated on the FP, and the prevalence showed a slightly decreasing trend (regression coefficient: −0.24), not statistically significant (*p*-value 0.16), resulting in an absence of correlation. Similar results were obtained when the incidence is considered. The regression analysis between sales volume and prevalence presented, as expected, a positive slope (regression coefficient: 0.41) but did not reach an acceptable level of significance (*p*-value: 0.026). Volumes observed include all the indications of the medicines, orphan and eventually not orphan, therefore, did not follow the prevalence trend (see Supplementary Panels 1, 2).

A comparison between data on prevalence and incidence collected from EPAR, COMP (22), Orphanet (21) and those presented by MAH in the P&R dossier was performed (see Supplementary Panel 3). For products for which both types of sources on prevalence (83%) and incidence (86%) were available, it emerged that the retrieved information were coherent (*R*^2^:1).

## Discussion

The introduction of strong incentives for orphan drug development allowed the discovery of several innovative therapies for rare diseases (23). However, the price of orphan drugs makes these products highly disruptive from a payer's perspective, posing serious challenges to the sustainability of health care systems, in particular for countries having a universalistic approach like Italy (24). The high cost of drug development and the small targeted population are often recalled as reasons for high prices of orphan drugs. Nevertheless, it was recently found that the clinical costs per approved orphan drug can be half that of a non-orphan drug (25) and a study having investigated the research and development spending for cancer drugs (9 of 10 were orphan drugs) showed that the mean and the median cost of development was lower than the revenues earned after the approval (in some cases the revenues were 10-fold higher than research and development spending) (26). Several orphan drugs developers can now benefit from extremely high revenues and in most cases high therapy costs are not correlated with the epidemiology of the related rare disease. This should stimulate debate on the development of new criteria on orphan drugs pricing, including the reshaping of the distribution of incentives at European and national level.

In general, the lack of alternative therapies tends to reduce payers' negotiating power to set the price of an orphan drug (19), intended for a small population of patients. Our findings suggested that the epidemiology of the rare disease influences marginally the cost of therapy of orphan drugs and it seems that the application of MEAs does not improve the correlation between the therapy cost with the prevalence/incidence since using the final price in place of ex-factory price, published on IOG results did not change.

With regards to the absence of correlation between rare disease epidemiology and sales volume of orphan drugs in the first year following a positive decision on P&R by AIFA, it should be noted that medicines, with an indication designated as orphan, often have other indications (orphan and not-orphan). Entering the market with an orphan designation benefitting from higher prices and then extending the indications, or “skillfully” selecting biomarkers to justify orphan or ultra-orphan developments (known as *salami-slicing*), are common commercial strategies which distort the original purpose of the legislation (22).

This mechanism is highly incentive for companies. It would be appropriate to separate the orphan indications from the non-orphan ones, as it already happens for medicines that have innovative indications compared to non-innovative ones.

The present study has some limitations. Firstly, it was based on a limited number of orphan drugs (*N* = 58) negotiated in the period 2014–2019, as data collection was not possible through AIFA's information systems prior to 2014. Furthermore, given that therapy cost was calculated just for reimbursed medicines, all non-reimbursed drugs (i.e., marketed with a free-price) and drugs undergoing P&R negotiation were not part of the statistical analysis, further reducing the dataset. However, such limitations are not expected to affect the statistical analysis although future studies conducted on larger datasets could further explore the validity of our findings. Another limitation could be the different sources used by industries to retrieve information on prevalence and incidence of rare diseases which, in some cases, do not refer to Italy.

It was not possible to use the estimates of patients eligible for the treatment, as provided by the company in the P&R dossiers (calculated generally on the basis of prevalence and incidence data) because no official data source could be used for their validation.

In many European countries Health Technology Assessment (HTA) is used to evaluate the value of new drug therapies, including orphan drugs. However, these methodologies have showed several limitations in defining common and universal recommendations (27). A clear rationale guiding the decision-making process for pricing and reimbursement is needed to make sustainable choices and preserve health care systems in the interest of patients. On the other hand, transparency on the price determinant is needed.

Health systems should perhaps consider the possibility to systematically renegotiate the price of orphan medicines when market shares become larger than they had been previously planned.

## Conclusions

Pricing is a complex process based on the assessment of multiple criteria. This study documented the absence of correlation between orphan drug cost as well as sales volume in the first year of marketing and the related rare disease prevalence/incidence in Italy.

In many cases, the availability of non-orphan indications, besides the first orphan indication of the drug, significantly increases the sale volumes of the product, which benefitted from numerous incentives available in the EU and at the national level.

## Data Availability Statement

The datasets presented in this article are not readily available because the price by single medicine is not possible to be disclaimed since confidential price agreement. Requests to access the datasets should be directed to a.cangini@aifa.gov.it.

## Author Contributions

FV, ADF, AP, and FT: conception and design. ADF, AP, FV, and DS: acquisition of data. FV and AC: drafting of the manuscript. ADF, AP, FV, FT, AC, AA, AG, DS, GTr, and GTa: critical revision of the manuscript for important intellectual content. ADF and AP: statistical analysis. All the authors interpretation of data. All authors contributed to the article and approved the submitted version.

## Author Disclaimer

The views expressed in this article are the personal views of the authors and may not be understood or quoted as being made on behalf of or reflecting the position of the respective authors' organizations.

## Conflict of Interest

FV, ADF, AP, AC, DS, and FT were employed by Agenzia Italiana del Farmaco (AIFA). GT was employed by Zorginstituut Nederland. The remaining authors declare that the research was conducted in the absence of any commercial or financial relationships that could be construed as a potential conflict of interest.

## Publisher's Note

All claims expressed in this article are solely those of the authors and do not necessarily represent those of their affiliated organizations, or those of the publisher, the editors and the reviewers. Any product that may be evaluated in this article, or claim that may be made by its manufacturer, is not guaranteed or endorsed by the publisher.
