# Supplementary material for: Orphan Drug Prices and Epidemiology of Rare Diseases: A Cross-Sectional Study in Italy in the Years 2014–2019
Source: Front Med (Lausanne). 2022 Feb 17;9:820757. doi: 10.3389/fmed.2022.820757 (PMC8891228; doi:10.3389/fmed.2022.820757)

**SUPPLEMENTARY MATERIAL**

**Table 1.** Overview of the ATC distribution of the orphan drugs that applied for P&R in Italy in the period 2014-2019

| **ATC** | **TOT** (89) | **Reimbursed** (N=58)  *65,2%* | **Not Reimbursed** (N=31)  *34,8%* |
| --- | --- | --- | --- |
| A - alimentary tract and metabolism | 16 | 9 | 2 (C) + 2 (Cnn) + 3 (negotiation not concluded) |
| B - blood and blood forming organs | 7 | 2 | 2 (C) + 1 (Cnn) + 2 (negotiation not concluded) |
| C - cardiovascular system | 4 | 2 | 1 (withdrawn) + 1 (negotiation not concluded) |
| D - dermatologicals | 2 | 1 | 1 (C) |
| J - antiinfectives for systemic use | 7 | 5 | 1 (C) + 1 (Cnn) |
| L - antineoplastic and immunomodulating agents | 38 | 31 | 3 (C) + 2 (Cnn) + 2 (negotiation not concluded) |
| M - musculo-skeletal system | 3 | 3 | 0 |
| N - nervous system | 3 | 1 | 1 (C) + 1 (negotiation not concluded) |
| R - respiratory system | 3 | 1 | 1 (C) + 1 (Cnn) |
| S - sensory organs | 5 | 3 | 2 (Cnn) |
| H – Systemic Hormonal Preparations, excl. Sex hormones and insulins | 1 | 0 | 1 (negotiation not concluded) |

*20 products are not in the list because they have not concluded the negotiation process yet (19) or have been withdrawn (1)*

**Table 2.** Distribution of the orphan drugs that concluded the negotiation process for P&R in Italy in the period 2014-2019 by ATC level and year

| **YEAR of P&R in ITA** (GU) | **TOT num of products** *(negotiation concluded -69-)* | **ATC**  **A** | **ATC**  **B** | **ATC**  **C** | **ATC**  **D** | **ATC**  **J** | **ATC**  **L** | **ATC**  **M** | **ATC**  **N** | **ATC**  **R** | **ATC**  **S** | **ATC**  **H** |
| --- | --- | --- | --- | --- | --- | --- | --- | --- | --- | --- | --- | --- |
| **2014** | 5 | 1 |  | 1 |  | 1 | 2 |  |  |  |  |  |
| **2015** | 8 | 1 | 1 | 1 |  | 1 | 3 |  |  | 1 |  |  |
| **2016** | 14 |  | 1 |  | 2 | 2 | 8 |  |  | 1 |  |  |
| **2017** | 19 | 4 | 1 |  |  | 1 | 7 | 1 | 4 |  | 1 |  |
| **2018** | 13 | 4 |  |  |  | 1 | 7 |  |  |  | 1 |  |
| **2019** | 10 |  | 1 |  |  |  | 7 | 1 |  |  | 1 |  |

**Figure 1.** Correlation analysis between the therapy-cost calculated on ex-factory price (published on IOG) and the therapy-cost calculated on the final price (FP)


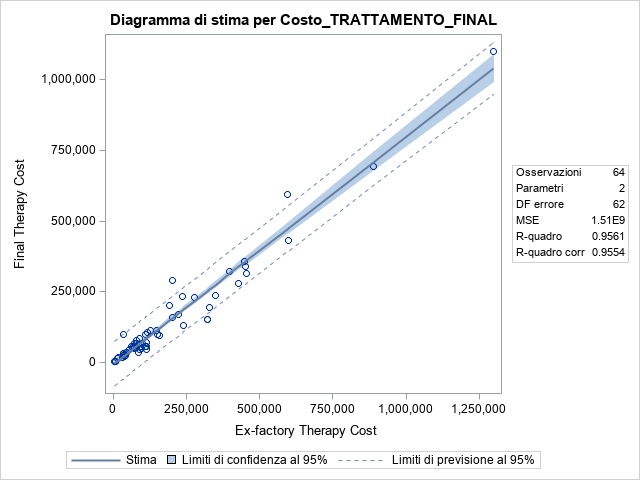


**Sensitivity analysis: incidence**

Figure 2. Correlation analysis between annual cost (calculated with ex-factory price) and incidence


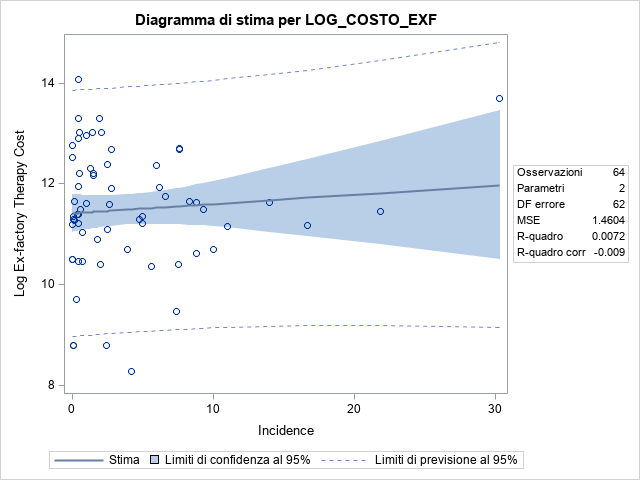


Figure 3. Correlation analysis between annual cost (calculated with final price) and incidence


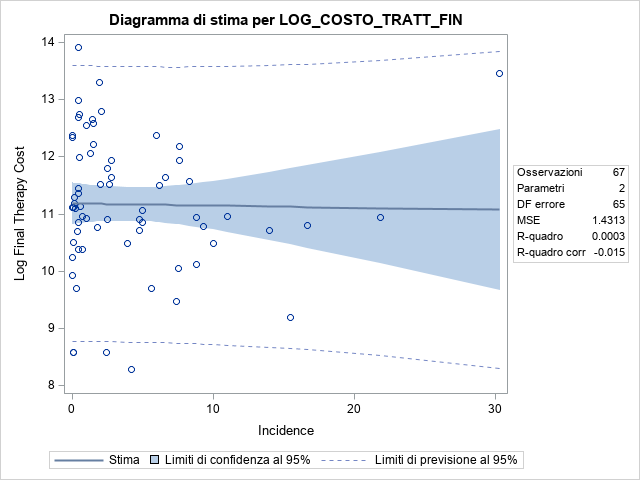


Figure 4. Correlation analysis between sales volume and incidence


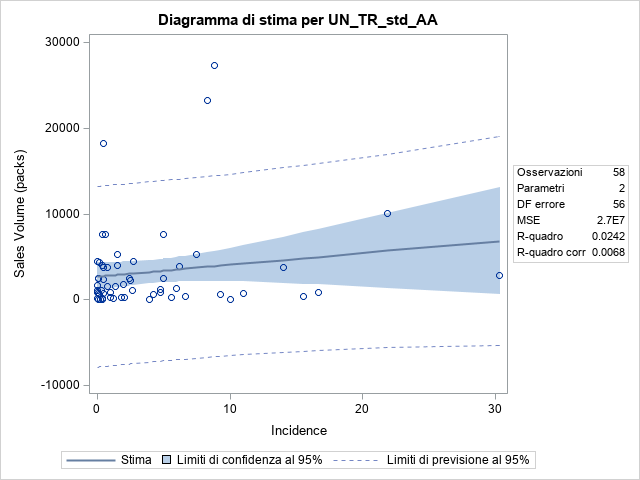


**Figure 5.** Correlation analysis between the log of the annual therapy cost (calculated on the FP) and the sales volume in the first year of commercialization in Italy.


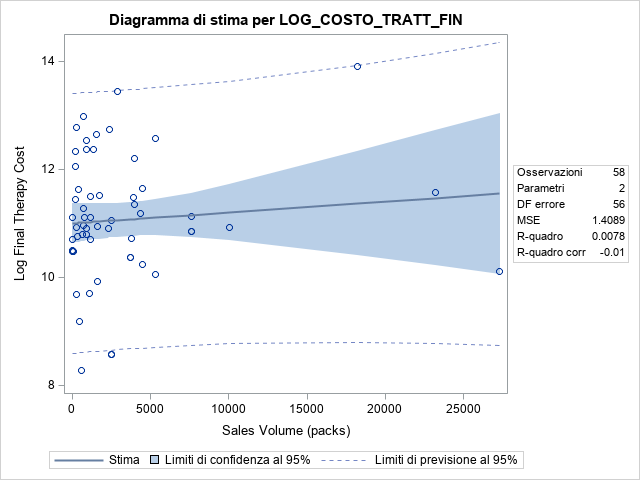


**Sensitivity analysis on antineoplastic and immunomodulating agents (ATC L)**

Table 3. Correlation analyses on on antineoplastic and immunomodulating agents (ATC L)

| **Pearson's correlation coefficients Prob > \|r\| sotto H0: Rho=0  Num. Of observations** | | | | | | | | |
| --- | --- | --- | --- | --- | --- | --- | --- | --- |
|  | **Sales Volume** | **Prevalence** | **Incidence** | **Ex factory price** | **Final price** | **Log of ex factory price** | **Log of final price** |  |
| **Sales Volume** | 1.00000  29 | 0.41262 0.0261 29 | 0.26473 0.1652 29 | -0.14717 0.4549 28 | -0.11479 0.5532 29 | -0.06712 0.7344 28 | -0.06167 0.7506 29 |  |
| **Prevalence** | 0.41262 0.0261 29 | 1.00000  36 | 0.68207 <.0001 36 | -0.28413 0.0981 35 | -0.25209 0.1380 36 | -0.21535 0.2141 35 | -0.24182 0.1554 36 |  |
| **Incidence** | 0.26473 0.1652 29 | 0.68207 <.0001 36 | 1.00000  36 | -0.27476 0.1102 35 | -0.30052 0.0749 36 | -0.19208 0.2690 35 | -0.27394 0.1059 36 |  |
| **Ex factory price** | -0.14717 0.4549 28 | -0.28413 0.0981 35 | -0.27476 0.1102 35 | 1.00000  35 | 0.94633 <.0001 35 | 0.84964 <.0001 35 | 0.85904 <.0001 35 |  |
| **Final price** | -0.11479 0.5532 29 | -0.25209 0.1380 36 | -0.30052 0.0749 36 | 0.94633 <.0001 35 | 1.00000  36 | 0.75494 <.0001 35 | 0.82783 <.0001 36 |  |
| **Log of ex factory price** | -0.06712 0.7344 28 | -0.21535 0.2141 35 | -0.19208 0.2690 35 | 0.84964 <.0001 35 | 0.75494 <.0001 35 | 1.00000  35 | 0.96485 <.0001 35 |  |
| **Log of final price** | -0.06167 0.7506 29 | -0.24182 0.1554 36 | -0.27394 0.1059 36 | 0.85904 <.0001 35 | 0.82783 <.0001 36 | 0.96485 <.0001 35 | 1.00000  36 |  |


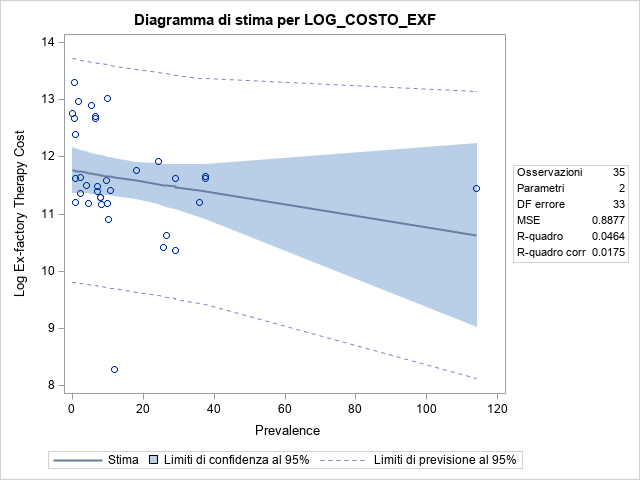

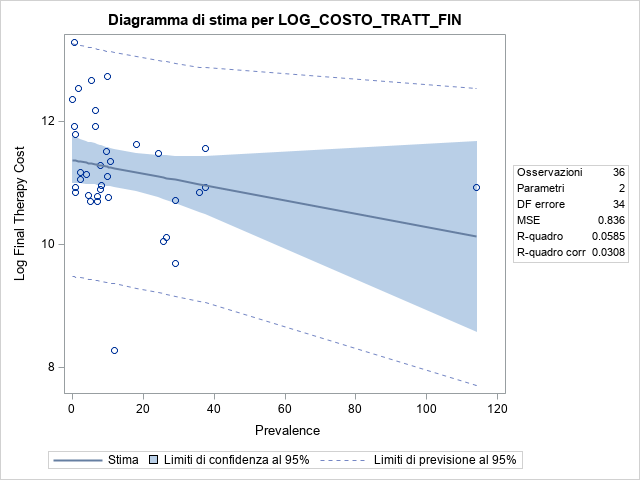

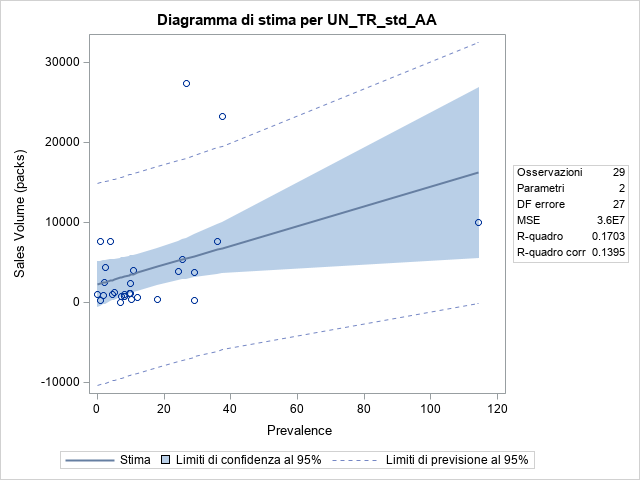

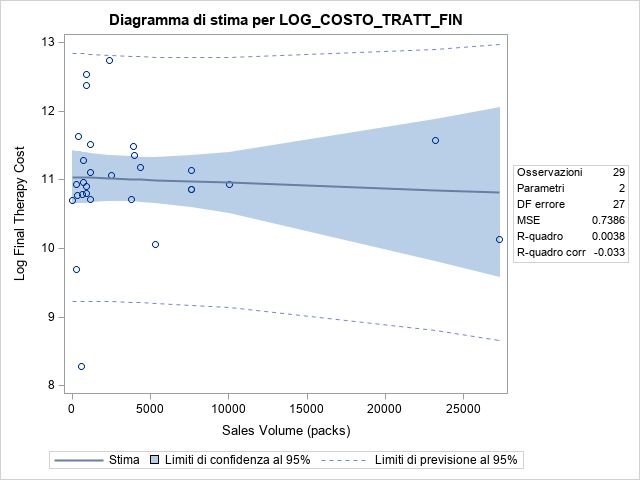

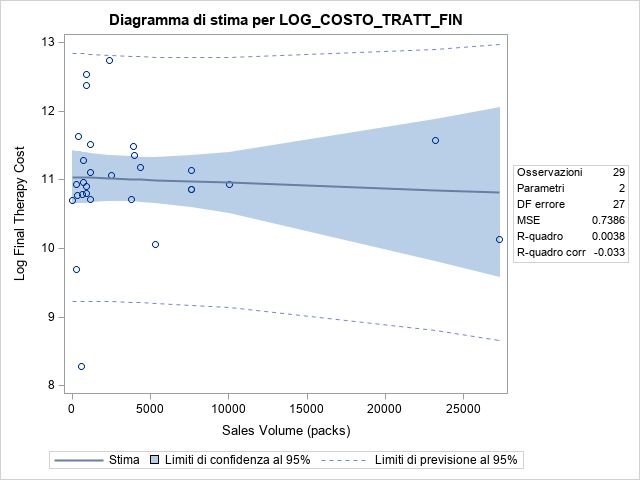


Panel 1. Sensitivity Analysis: ATC L


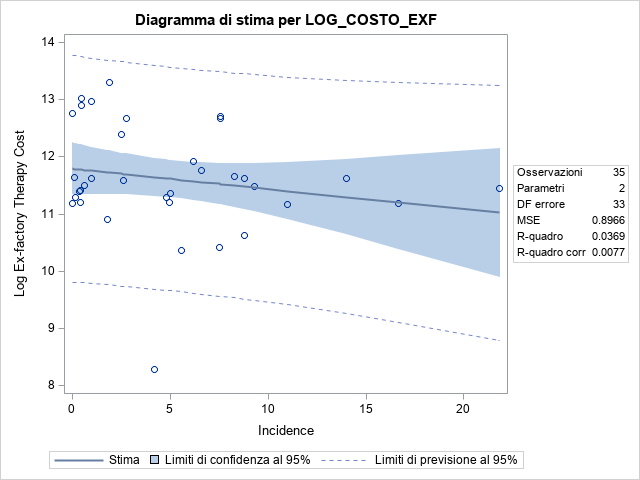

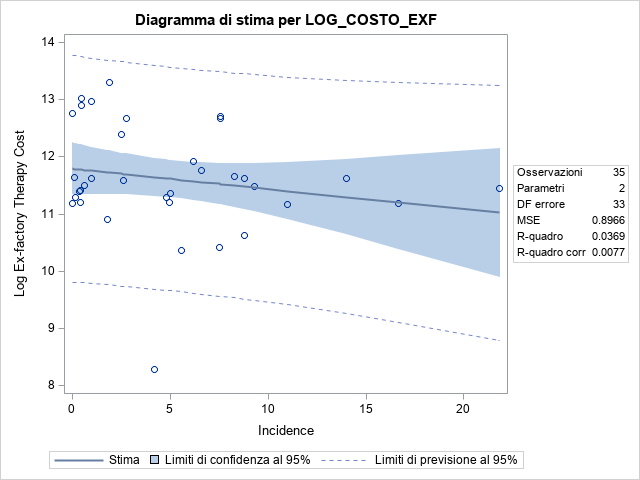

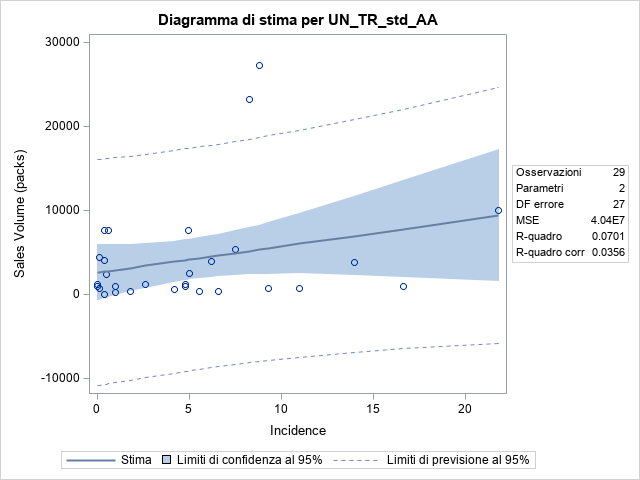


Panel 2. Sensitivity analysis ATC L /incidence

**COMPARISION BETWEEN PREVALENCE & INCIDENCE DATA FROM DOSSIER AND FROM EPAR/COMP/ORPHANET**

**Panel 3**

- 1. **Prevalence from EPAR/COMP/Orphanet vs Prevalence from Dossier**


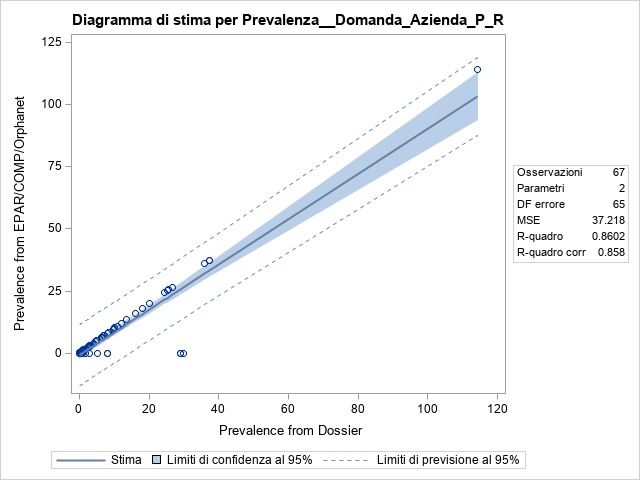


- 1. **Incidence from EPAR/COMP/Orphanet vs Incidence from Dossier**


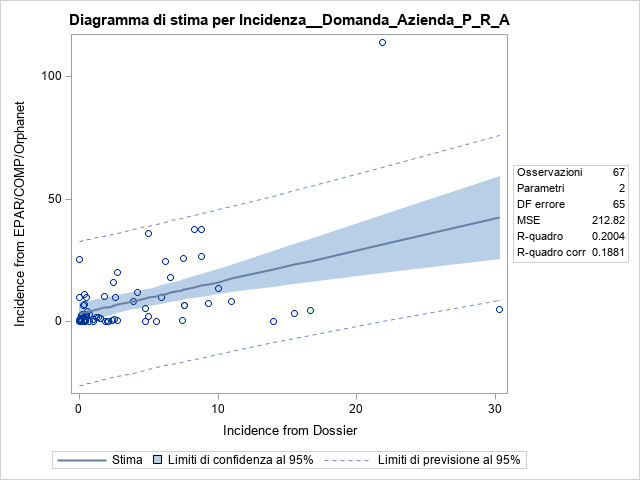

Supplement: Supplementary file 1 [file Data_Sheet_1.docx]
